# Presumed Amniotic Fluid Embolism Complicated by Disseminated Intravascular Coagulation and Refractory Postpartum Hemorrhage: A Case Report and Narrative Review

**DOI:** 10.3390/life16071207

**Published:** 2026-07-21

**Authors:** Yasmin Schäffter, David Schmidbauer, José Valles Fons, Helena Schäffter, Jonah Bosserhoff, Erika-Gyöngyi Bán

**Affiliations:** 1 Department of Clinical Pharmacology, Medical Campus Hamburg (UMCH), George Emil Palade University of Medicine, Pharmacy, Science and Technology of Târgu Mureș, 22761 Hamburg, Germany; david.schmidbauer@freenet.de (D.S.); josevallesfons@gmail.com (J.V.F.); helena.schaeffter@gmail.com (H.S.); 2 Faculty of Medicine, University of Duisburg-Essen, 45117 Essen, Germany; jonahbosserhoff25@gmail.com; 3 Department of Clinical Pharmacology, George Emil Palade University of Medicine, Pharmacy, Science, and Technology of Târgu Mureș, 540142 Târgu Mureș, Romania; erika.ban@umfst.ro

**Keywords:** amniotic fluid embolism, disseminated intravascular coagulation, postpartum hemorrhage, cesarean delivery, massive transfusion, peripartum cardiac arrest, case report

## Abstract

Amniotic fluid embolism (AFE) is a rare but catastrophic obstetric emergency characterized by sudden cardiorespiratory collapse, disseminated intravascular coagulation (DIC), and a high case-fatality rate. Because no confirmatory test exists, the diagnosis remains clinical and one of exclusion. We report a case of presumed AFE in a 39-year-old primigravida with uterine myomas, obesity, and chronic hypertension who underwent an elective primary cesarean delivery under spinal anesthesia. During manipulation of the placenta, the patient developed abrupt cardiovascular collapse requiring cardiopulmonary resuscitation, with return of spontaneous circulation followed by profound coagulopathy and refractory uterine atony. Management included goal-directed transfusion within a massive transfusion protocol, uterotonic therapy, a failed B-Lynch suture, supracervical hysterectomy, and a subsequent right oophorectomy for an ovarian-vein hemorrhage identified on imaging. Laboratory studies demonstrated an overt consumptive coagulopathy consistent with the International Society on Thrombosis and Haemostasis (ISTH) criteria, while a normal serum tryptase argued against an anaphylactic mechanism. The neonate was delivered in good condition (Apgar scores 9, 10, and 10 at 1, 5, and 10 min; umbilical-artery pH 7.38) and required no neonatal intensive care. The mother achieved full hemodynamic and neurological recovery. This case illustrates that survival from presumed AFE is achievable through early recognition, high-quality resuscitation, prompt correction of coagulopathy, and decisive surgical hemostasis, and it highlights the diagnostic reasoning required to distinguish AFE from its principal differential diagnoses.

## 1. Introduction

Amniotic fluid embolism (AFE) is one of the most feared complications of pregnancy and the peripartum period. It is classically defined by the abrupt onset of cardiorespiratory collapse, hypoxia, and disseminated intravascular coagulation (DIC), typically occurring during labor, delivery, or the immediate postpartum interval [1,2]. Despite advances in obstetric and critical care, AFE remains a leading direct cause of maternal mortality in high-income countries [3,4,5,6,7].

The pathophysiology is incompletely understood. Rather than a purely mechanical embolic event, current models describe an aberrant maternal immune and inflammatory response to fetal antigens entering the maternal circulation, triggering pulmonary vasoconstriction, right-heart strain, and a coagulation cascade that culminates in consumptive coagulopathy [1,8,9]. Because of this resemblance to anaphylaxis, the term “anaphylactoid syndrome of pregnancy” has also been used [10].

There is no laboratory or imaging test that confirms AFE; the diagnosis is clinical and is reached only after exclusion of other causes of peripartum collapse and hemorrhage [11,12]. For this reason, contemporary publications increasingly favor cautious, explicitly presumptive terminology. We adopt that convention throughout this report and refer to a presumed diagnosis of AFE. We describe the clinical course, diagnostic reasoning, and multidisciplinary management of a patient who survived presumed AFE complicated by overt DIC and refractory postpartum hemorrhage, and we situate the case within a narrative review of the contemporary literature.

## 2. Case Presentation

### 2.1. Patient and Intraoperative Course

A 39-year-old primigravida was admitted for an elective primary cesarean delivery. Her medical history was notable for class III obesity (body-mass index 40 to <50 kg/m^2^), chronic essential hypertension, gestational diabetes mellitus and a multinodular myomatous uterus. The procedure was performed under spinal anesthesia. A healthy female neonate was delivered, weighing 3690 g, with a length of 52 cm and a head circumference of 35.5 cm. Apgar scores were 9, 10, and 10 at 1, 5, and 10 min, respectively, and the umbilical-artery pH was 7.38. The neonate required no neonatal intensive care and remained with the family.

Shortly after delivery, during manipulation and removal of the placenta, the patient developed sudden cardiovascular collapse. Placental removal was technically difficult because of dense adherence to multiple uterine myomas; however, intraoperative findings did not confirm placenta accreta spectrum. Cardiopulmonary resuscitation (CPR) was initiated immediately. Return of spontaneous circulation (ROSC) was achieved after approximately 3 min of resuscitation. Oxygen saturation at the moment of collapse could not be reliably retrieved from the intraoperative record; the lowest documented value was 91%. Arterial blood gas sampling at the exact moment of cardiovascular collapse was not feasible because immediate resuscitative measures took priority. Consequently, the severity of respiratory compromise at symptom onset cannot be retrospectively quantified. The constellation of abrupt collapse during delivery, the absence of an alternative explanation, and the rapidly evolving coagulopathy described below prompted a presumptive working diagnosis of AFE, pending exclusion of other causes.

**Figure 1:**
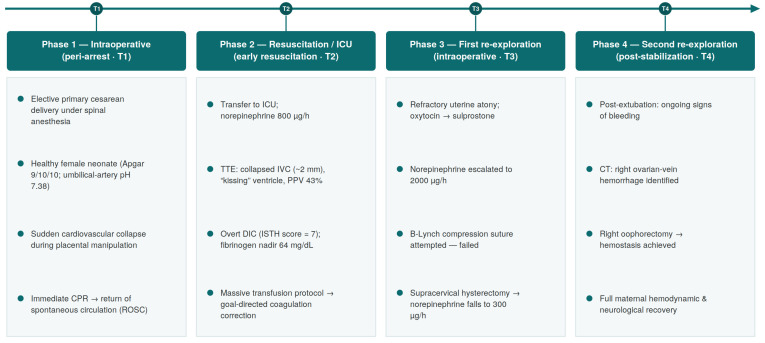
Clinical timeline of the management course, organized as a phase swimlane across the four management phases. Events are arranged on a neutral relative phase axis (T1–T4) reflecting the clinical sequence; no calendar times or dates are shown. CPR, cardiopulmonary resuscitation; CT, computed tomography; DIC, disseminated intravascular coagulation; ICU, intensive care unit; ISTH, International Society on Thrombosis and Haemostasis; IVC, inferior vena cava; PPV, pulse-pressure variation; ROSC, return of spontaneous circulation; TTE, transthoracic echocardiography.

Following ROSC, the patient was transferred to the intensive care unit (ICU). Norepinephrine was required at an initial rate of 800 µg/h to maintain perfusion. The clinical course was dominated by uterine atony and progressive hemorrhage, managed as detailed below. An overview of the entire management course is presented in Figure 1.

### 2.2. Echocardiographic and Hemodynamic Findings

Point-of-care transthoracic echocardiography (TTE) performed in the ICU demonstrated a collapsed inferior vena cava (diameter approximately 2 mm) and “kissing” ventricular walls consistent with marked hypovolemia, with a pulse-pressure variation of 43%; no pericardial effusion was seen. A formal cardiology-performed echocardiogram showed normal left-ventricular function, no evidence of right-ventricular strain or dilatation, and only mild mitral regurgitation. Cardiac troponin T peaked at 319 ng/L and declined thereafter, a pattern interpreted as demand ischemia in the setting of cardiac arrest and profound hemorrhagic shock rather than primary myocardial injury. D-dimers were markedly elevated. Serum tryptase was obtained to evaluate for an anaphylactic mechanism.

It should be noted that, although right-ventricular dysfunction is a characteristic early echocardiographic feature of AFE, no right-ventricular strain was captured on the available studies. This is most plausibly explained by the timing of imaging, which was performed after resuscitation and volume resuscitation rather than at the moment of collapse; this limitation is addressed explicitly in the Section 6.

### 2.3. Mapping to Diagnostic Criteria

The presentation was mapped against the research case-definition criteria proposed by the Society for Maternal-Fetal Medicine (SMFM) and Clark et al. [2,13]. (i) Sudden cardiorespiratory arrest or acute hypotension occurred during delivery. (ii) Overt DIC, as defined by the International Society on Thrombosis and Haemostasis (ISTH) scoring system, developed in the immediate aftermath (see below). (iii) The onset occurred during the cesarean delivery, within the time window specified by the criteria. (iv) There was no fever (temperature ≥ 38 °C). Oxygen saturation data at the exact moment of collapse were incomplete due to the emergency circumstances. The lowest documented value in the available anesthetic record was 91%.

Arterial blood gas analyses obtained during the post-resuscitation phase demonstrated severe metabolic acidosis consistent with profound circulatory shock. The lowest recorded pH was 7.14, with a base excess of −14.1 mmol/L, a bicarbonate concentration of 15.0 mmol/L, and a peak lactate concentration of 5.9 mmol/L. Available measurements demonstrated preserved oxygenation (PaO_2_ 121–241 mmHg; oxygen saturation 99–100%). At a documented FiO_2_ of 35%, the PaO_2_/FiO_2_ ratio was 346, indicating no relevant impairment of oxygenation. However, arterial blood gas measurements from the exact moment of cardiovascular collapse were unavailable; therefore, the presence and severity of respiratory compromise at symptom onset cannot be determined with certainty.

On balance, the presentation was considered highly consistent with the SMFM/Clark research definition, although incomplete documentation of oxygenation at the moment of collapse represents a limitation.

### 2.4. Coagulation Profile and DIC Scoring

**Figure 2:**
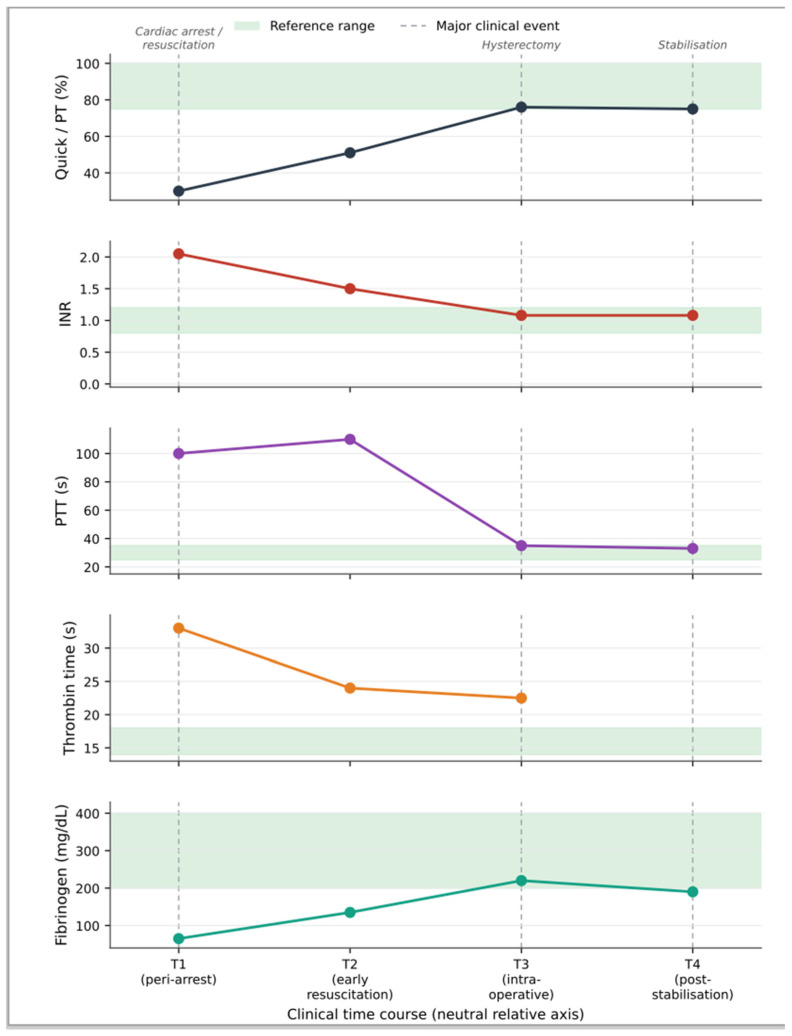
Temporal evolution of coagulation parameters during resuscitation and surgical management. Values are plotted on a neutral relative time axis (T1–T4) reflecting the clinical sequence rather than calendar time. Shaded bands indicate the local reference range for each parameter; dashed vertical lines mark major clinical events. All parameters were most deranged at the peri-arrest time point (T1) and improved with goal-directed substitution. Full numerical values are listed in Supplementary Table S1.

Serial coagulation testing documented a severe but ultimately reversible consumptive coagulopathy. The most deranged values were recorded at the peri-arrest time point and improved progressively with goal-directed substitution. The prothrombin activity (Quick) fell to a nadir of 29% (reference > 74%), corresponding to an international normalized ratio (INR) of 2.1 (reference < 1.2); the activated partial thromboplastin time (PTT) was prolonged to 100 s and transiently to 113 s (reference 23.9–33.2 s); the thrombin time peaked at 33.2 s (reference 14.4–19.0 s); and the fibrinogen concentration reached a nadir of 64 mg/dL (reference 193–412 mg/dL). With substitution, all parameters recovered toward the reference range over the subsequent time points, fibrinogen rising to 134, then 223, and 189 mg/dL. The full serial profile is provided in Supplementary Table S1 and illustrated in Figure 2.

Applying the ISTH scoring system at the peri-arrest nadir yielded an aggregate score in the overt-DIC range (approximately 7 points): a platelet count of 88 × 10^9^/L (1 point), a strong elevation of D-dimer (3 points), a fibrinogen nadir below 100 mg/dL (1 point), and a markedly prolonged prothrombin time corresponding to a Quick of 29%/INR 2.1 (2 points). A score ≥ 5 is consistent with overt DIC, supporting the second SMFM criterion. Importantly, serum tryptase, drawn separately, was within the normal range (<11.0 µg/L), arguing against an anaphylactic mechanism, although a normal serum tryptase cannot completely exclude anaphylaxis, as discussed in Section 4.2.

### 2.5. Surgical and Transfusion Management

Uterine atony was treated initially with oxytocin and subsequently with sulprostone (Nalador). As hemorrhage continued, norepinephrine was escalated to 2000 µg/h. A first surgical re-exploration revealed a persistently atonic uterus; a B-Lynch compression suture was attempted but failed to achieve hemostasis, and a supracervical hysterectomy was therefore performed. A massive transfusion protocol was activated, with goal-directed correction of coagulopathy. With surgical and hematologic control of bleeding, the norepinephrine requirement fell to 300 µg/h, reflecting improving hemodynamic stability.

Approximately 24 h after hysterectomy, ongoing signs of bleeding prompted a second surgical re-exploration. Computed tomography identified a hemorrhage from the right ovarian vein, which was managed with a right oophorectomy. The patient subsequently stabilized. Cumulative transfusion and hemostatic therapy during the entire episode are summarized in Table 1. In total, the patient received 18 units of packed red blood cells, 16 units of fresh frozen plasma, 2 units of lyophilized plasma, and 2 platelet concentrates. Tranexamic acid (1 g intravenously) was administered within the first hour of hemorrhage.

## 3. Narrative Review/Background

This section provides a narrative review of the contemporary literature to contextualize the present case. It is not a systematic review: no formal protocol, PRISMA flow, or quantitative synthesis was undertaken. We searched English- and German-language sources published between 2013 and 2025, prioritizing population-based cohorts, society guidelines, and recent reviews, and selected references by relevance rather than by predefined eligibility criteria.

### 3.1. Epidemiology

AFE is rare, and reported incidence varies with case definition, ascertainment method, and population. Estimates generally fall in the range of approximately 2–8 per 100,000 deliveries, with validated population-based cohorts clustering at 1.9–6.1 per 100,000 [4,5,14]. A recent 20-year analysis of United States data reported a mean incidence of approximately 4.9 per 100,000 deliveries and a mean case-fatality rate of 17.7% [15]. Across studies, reported case-fatality estimates span a wide range—broadly 11–44%—reflecting differences in diagnostic stringency and the inclusion of non-fatal cases [3,4,5]. Recognized associations include advanced maternal age, multiparity, cesarean and operative delivery, placental abnormalities, and medical induction of labor, although AFE also occurs in the absence of identifiable risk factors [4,5,11]. Reported risk factors are best understood as epidemiologic associations rather than as causal or predictive determinants [12].

### 3.2. Pathophysiology

Contemporary models conceptualize AFE as an abnormal host response to fetal antigenic material entering the maternal circulation, rather than as a simple mechanical obstruction. Activation of inflammatory and complement pathways—sometimes described as an “immune storm”—is accompanied by a procoagulant “coagulation storm” driven by tissue-factor–rich amniotic constituents, producing pulmonary vasoconstriction, transient right-heart strain, and consumptive coagulopathy [1,8,9]. The phenotypic overlap with anaphylaxis underlies the alternative designation as the anaphylactoid syndrome of pregnancy [10].

### 3.3. Diagnosis and the Role of Echocardiography

Because no confirmatory test exists, diagnosis rests on the clinical syndrome and the exclusion of alternatives, supported in research settings by the SMFM/Clark criteria [2,13,16,17]. Echocardiography may demonstrate acute right-ventricular dysfunction and pulmonary hypertension in the early phase, and serial imaging can document the subsequent transition to a left-sided and vasodilatory picture [18]. Findings depend heavily on the timing of imaging relative to the index event, a point directly relevant to the present case.

### 3.4. Management Principles

Management is supportive and multidisciplinary, centering on high-quality resuscitation, early and aggressive correction of coagulopathy, and control of obstetric hemorrhage [16,19,20,21]. Massive transfusion protocols and goal-directed, laboratory-guided hemostatic therapy are central to contemporary practice [22].

#### Management of Uterine Atony and Postpartum Hemorrhage

The management of refractory uterine atony and postpartum hemorrhage in this case followed established stepwise guideline algorithms and is therefore summarized here only briefly. Treatment escalates from uterotonic pharmacotherapy through uterine-sparing surgical measures (including compression sutures and arterial ligation) to hysterectomy when conservative measures fail, as set out in the German AWMF S2k guideline (registry no. 015/063) and in the corresponding RCOG and ACOG recommendations [23,24,25]. Readers are referred to these guidelines for the detailed algorithm.

## 4. Discussion

This case illustrates survival from presumed AFE complicated by overt DIC and refractory postpartum hemorrhage. Several features merit discussion: the diagnostic reasoning that led to a presumptive diagnosis, the differential diagnoses that had to be excluded, and the pharmacologic considerations surrounding hemostatic therapy.

### 4.1. Diagnostic Reasoning

The temporal association between placental manipulation, abrupt cardiovascular collapse, and the rapid development of overt DIC, in the absence of an alternative explanation, is the central argument supporting a presumed diagnosis of AFE. The diagnosis remains one of exclusion, and we have deliberately retained presumptive terminology throughout. The coherence of the coagulation data—uniformly most deranged at the peri-arrest time point and improving with substitution—supports a single, hyperacute consumptive process rather than a slowly evolving coagulopathy.

### 4.2. Differential Diagnosis

Several conditions can mimic AFE and were considered:

Local anesthetic systemic toxicity (LAST). LAST was the initial working hypothesis recorded at ICU admission. However, the temporal profile and the early, severe consumptive coagulopathy are atypical for LAST, which is not characteristically associated with overt DIC. This diagnosis was therefore considered unlikely on cautious review.

Anaphylaxis. Given the phenotypic overlap between AFE and anaphylaxis, a serum tryptase was obtained. The result was within the normal range (<11.0 µg/L), which argues against an anaphylactic mechanism. Total serum IgE was 2.4 kU/L.

Pulmonary embolism. Acute pulmonary thromboembolism was considered. Although echocardiography did not demonstrate right-ventricular strain, imaging was performed after resuscitation and volume replacement, which may have limited the detection of transient right-heart dysfunction. Nevertheless, the overall clinical presentation was considered atypical for primary pulmonary thromboembolism.

Peripartum cardiomyopathy and myocardial infarction. Biventricular function was preserved on formal echocardiography, and the troponin T rise (peak 319 ng/L, declining) was interpreted as demand ischemia in the context of cardiac arrest and hemorrhagic shock rather than primary myocardial disease.

Sepsis. The hyperacute onset and the absence of fever were inconsistent with septic shock; a procalcitonin of 2.4 ng/mL measured later was attributed to the post-resuscitation inflammatory state.

Other obstetric causes. Placental abruption, eclampsia, and uterine rupture were not supported by the intraoperative findings or the clinical course.

### 4.3. Pharmacologic Considerations

Tranexamic acid. Tranexamic acid was administered as part of guideline-consistent management of obstetric hemorrhage [26,27,28,29]. Given the simultaneous use of multiple hemostatic interventions and surgical hemostasis, the independent contribution of tranexamic acid to the outcome cannot be isolated in a single case; its use was consistent with current guideline recommendations.

Recombinant activated factor VII. Recombinant activated factor VII has been described in refractory AFE-associated hemorrhage but carries a recognized thrombotic risk, and the available evidence is limited to case reports [30]. It is best reserved for salvage situations after standard measures have failed.

Norepinephrine. In this case, the norepinephrine requirement tracked the hemorrhagic course—escalating from 800 to 2000 µg/h during active bleeding and falling to 300 µg/h once surgical and hematologic control was achieved—and thus served as a useful bedside marker of hemorrhage control.

### 4.4. Comparison with the Literature

The clinical trajectory of this patient is consistent with previously reported survivor cases in which early recognition, aggressive transfusion, and timely surgical hemostasis were decisive [18,30,31]. The novelty of this case lies in the combination of intraoperative cardiac arrest, refractory uterine atony requiring hysterectomy, and a delayed ovarian-vein hemorrhage requiring oophorectomy, all with full maternal recovery—a constellation with very few published precedents.

## 5. Conclusions

This case demonstrates that survival with full maternal and neurological recovery is achievable in presumed AFE complicated by overt DIC and refractory postpartum hemorrhage. In this patient, the decisive interventions were prompt high-quality CPR with rapid ROSC, activation of a massive transfusion protocol with goal-directed correction of coagulopathy, timely progression to supracervical hysterectomy after a failed B-Lynch suture, and vigilant postoperative monitoring that identified and controlled a delayed ovarian-vein hemorrhage. As a single case, these observations are illustrative rather than generalizable, and they should be interpreted as supporting—not establishing—the value of early recognition and coordinated multidisciplinary management in this rare and unpredictable emergency.

## 6. Limitations

Several limitations should be acknowledged. First, AFE remains a clinical diagnosis of exclusion, and no confirmatory test was available; the diagnosis is therefore presumptive. Second, characteristic early right-ventricular dysfunction was not captured on echocardiography, most plausibly because imaging was performed after resuscitation and volume resuscitation rather than at the moment of collapse; the absence of this finding does not exclude AFE but is reported transparently. In addition, end-tidal carbon dioxide (ETCO_2_) measurements at the time of collapse were unavailable, limiting retrospective assessment of respiratory compromise during the initial event. Finally, as a single case report, the findings cannot be generalized and are intended to complement, not substitute for, higher-level evidence.

## Figures and Tables

**Table 1:** Cumulative transfusion and hemostatic therapy across the management phases. Quantities are totals for the episode; “Yes”/”—“ indicate whether a product was administered during a given phase.

Product/Therapy	Total	Resuscitation/ICU	Hysterectomy	Re-Exploration
Packed red blood cells	18 units	Yes	Yes	Yes
Fresh frozen plasma	16 units	Yes	Yes	Yes
Lyophilized plasma	2 units	Yes	—	—
Platelet concentrate	2 units	Yes	Yes	—
Tranexamic acid	1 g IV	Yes	—	—

ICU, intensive care unit.

## Data Availability

The data presented in this study are not publicly available due to patient privacy and data protection restrictions.

## Supplementary Materials

The following supporting information can be downloaded at: https://www.mdpi.com/article/10.3390/life16071207/s1, Table S1: Serial coagulation profile during resuscitation and surgical management.

## Abbreviations

Abbreviation	Definition
ACOG	American College of Obstetricians and Gynecologists
AFE	amniotic fluid embolism
AWMF	Association of the Scientific Medical Societies in Germany
CPR	cardiopulmonary resuscitation
DIC	disseminated intravascular coagulation
ICU	intensive care unit
INR	international normalized ratio
ISTH	International Society on Thrombosis and Haemostasis
LAST	local anesthetic systemic toxicity
PTT	partial thromboplastin time
RCOG	Royal College of Obstetricians and Gynaecologists
ROSC	return of spontaneous circulation
SMFM	Society for Maternal-Fetal Medicine
TTE	transthoracic echocardiography
