# Supplementary material for: Presumed Amniotic Fluid Embolism Complicated by Disseminated Intravascular Coagulation and Refractory Postpartum Hemorrhage: A Case Report and Narrative Review
Source: Life (Basel). 2026 Jul 21;16(7):1207. doi: 10.3390/life16071207 (PMC13413040; doi:10.3390/life16071207)
Supplement: Supplementary file 1 [file life-16-01207-s001.zip › life-4377479-supplementary.pdf]

Supplementary Materials

Supplementary Table S1. *Serial coagulation profile during resuscitation and surgical management, shown on a neutral relative time axis (T1–T4). A dash indicates a parameter not sampled at that time point. Serum tryptase, drawn on a separate occasion, was within the normal range (<11.0 μg/L), arguing against an anaphylactic mechanism.*

Time point	Quick / PT (%)	INR	PTT (s)	Thrombin time (s)	Fibrinogen (mg/dL)
T1 (peri-arrest)	29	2.1	100	33.2	64
T2 (early resuscitation)	51	1.5	113	23.9	134
T3 (intraoperative)	76	1.1	34.3	22.4	223
T4 (post-stabilisation)	75	1.1	33.2	–	189
Reference range	*>74*	*<1.2*	*23.9–33.2*	*14.4–19.0*	*193–412*

PT, prothrombin time; INR, international normalised ratio; PTT, partial thromboplastin time. Time points are shown on a neutral relative axis (T1–T4) reflecting the clinical sequence. A dash indicates a parameter not sampled at that time point. Serum tryptase, drawn on a separate occasion, was within the normal range (<11.0 μg/L), arguing against an anaphylactic mechanism.
